# Post-infarction ventricular fibrillation mechanisms: Insights from combined body surface potential mapping and late gadolinium-enhanced CMR

**DOI:** 10.1186/1532-429X-18-S1-P198

**Published:** 2016-01-27

**Authors:** Hubert Cochet, Stephanie Clement-Guinaudeau, Marjorie Salel, Olivier Corneloup, Michel Montaudon, François Laurent

**Affiliations:** IHU Liryc - CHU / Université de Bordeaux, Pessac, France

## Background

The mechanisms involved in the sustenance of scar-related ventricular fibrillation (VF) are poorly understood. Recent developments in body surface potential mapping (BSM) now enable non-invasive, real-time and whole-heart assessment of cardiac activation during VF. We sought to analyze the relationships between focal/reentrant activities during VF and scar characteristics on late gadonlinium-enhanced (LGE) CMR in patients with ischemic VF.

## Methods

7 patients with history of prior myocardial infarction who presented a VF episode underwent CMR prior to ICD implantation. LGE imaging was performed on a 1.5T system (Avanto, Siemens Medical Solutions, Erlangen, Germany) 15 min after the injection of 0.2 mmol/Kg gadoterate meglumine, using an inversion recovery-prepared and respiratory navigated 3D Turbo FLASH sequence with fat saturation, in order to acquire a whole heart volume at high spatial resolution (pixel size 1.25 × 1.25 ×2.5 mm). After ICD implant, a 252-electrode vest was positioned on the patient's thorax in order to perform BSM during several seconds of VF, as induced during ICD testing. Dedicated algorithm was used to reconstruct unipolar electrograms from body surface recordings on a biventricular epicardial geometry. Reentry was defined on phase maps as any phase singularity lasting more than 4 rotations. Focal activity was defined as any site exhibiting repeated focal activation (at least 2 consecutive discharges). On LGE images, the myocardium was manually contoured and scar was segmented using automated adaptive histogram thresholding. The location of this substrate was projected onto a biventricular epicardial segmentation. This biventricular geometry from imaging was registered to the one from BSM using an iterative closest point algorithm. The geodesic distance to the closest scar border was computed for each point of the mesh, expressed in mm. On these distance maps, negative values indicate areas within scar, positive values areas outside scar, and zero value indicates scar border.

## Results

Scar location was anterior in 4 patients, inferior in 2 and lateral in 1. Scar area was 76 [IQR 53-85] cm^2^. LVEF was 33 [IQR 25-39] %. The median number of reentrant regions was 4 [IQR 3-4.5] per patient, and the median number of focal sites was 4 [IQR 3.5-6.5] per patient. Reentrant regions clustered to scar borders, with a distance to scar in regions with vs without reentry of +3.6 [-7.1 - +20.0] vs +28.2 [+6.9 - +50.5] mm, p < 0.0001. In contrast, sites exhibiting focal activity did not relate to scar location, with a distance to scar in regions with vs without focal activity of +36.5 [+24.7 - +71.9] vs +28.1 [+6.8 - +50.3] mm, P=0.47.

## Conclusions

Post-infarction VF is driven by a combination of reentrant and focal sources. Reentrant activity clusters to scar borders, while focal activity does not relate to scar location. These results provide new insights into VF mechanisms, and pave the way for potential CMR-guided therapies aiming at substrate-modification in scar-related VF.

**Figure 1:**
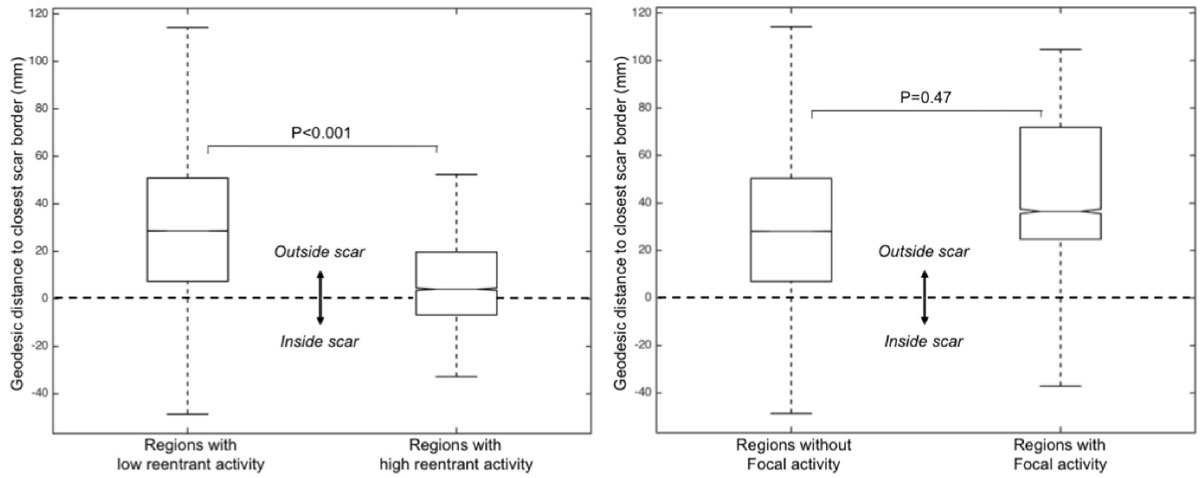
**Distribution of reentrant and focal activities during VF with respect to scar location**.

**Figure 2:**
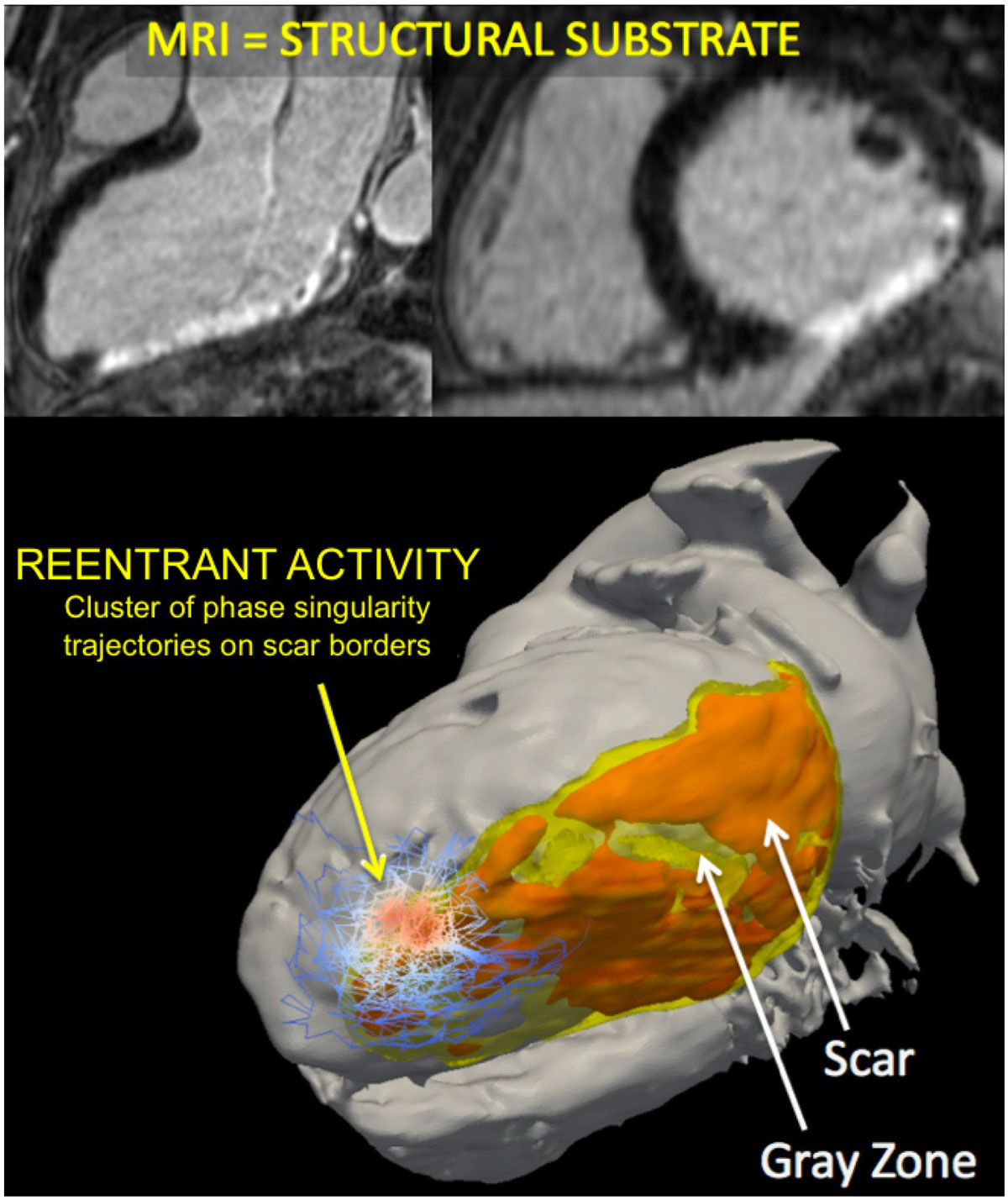
**62 year-old man with post-infarction VF. Reentrant activity clusters to scar borders**.

